# The Story of the Insane from Year to Year

**Published:** 1894-03-10

**Authors:** 


### THE STORY OF THE INSANE FROM YEAR TO YEAR.

(Sixth Series.)

### FIFE AND KINROSS ASYLUM.

The numbers in this asylum increased by eight during the year, there being on the books the names of 448 patients. There were 112 admissions, 51 recoveries, and 29 deaths. The recoveries therefore stand at 34·86 per cent. of the admissions, and the deaths at 6·41 per cent. of the average number resident. In 25 of the admissions the insanity was caused by drink, and in 33 by hereditary influences, while "previous attacks" is given as the assigned cause in 32. Fifteen of the deaths were due to some kind of brain disease, and only two to phthisis.

Dr. Turnbull tells us that he had under treatment at the same time a husband and wife. The latter seems to have been puerperal, and in the former the mental disease was brought on by anxiety at his wife's illness. Fortunately, says Dr. Turnbull, they both made good and rapid recoveries. Yes; it is fortunate in one sense; but what about the next generation?

The training of attendants has been continued during the year, and six have obtained the certificates of the Medico-psychological Association. The visitors say that the Medical Superintendent has given complete satisfaction to the board.

The Commissioners say that "in view of the difficulties under which it is conducted, the management is very satisfactory and successful, and discloses much ability and conscientiousness on the part of Dr. Turnbull." It would seem that eighteen attendants resigned, and two were dismissed during the year.

### DUNDEE ROYAL ASYLUM.

The numbers increased from 360 to 386. There were 188 admissions, 64 recoveries, and 42 deaths. The recoveries were 34 per cent. on the admissions, and the deaths 11·26 on the average number resident. Hereditary influence caused the insanity in 58 cases, and drink in 33. Brain diseases caused death in 27 cases, and consumption in 1. Dr. Rorie thinks that about 50 per cent. of the insane may be looked after either in the lunatic wards of the workhouses or in private dwellings, and thus may be relieved the ever-increasing pressure on asylum space. In Scotland this is no doubt practicable, but we doubt whether anything like 50 per cent. of our asylum patients could be so treated in or near our large manufacturing towns.

The weekly charge for paupers is from 11s. to 12s. 6d. The private patients pay from £25 to £180 a year.

### THREE COUNTIES ASYLUM (BEDFORD, HERTFORD, AND HUNTINGDON).

On January 1st this asylum contained 1,024 patients, and by the end of the year the number had risen to 1,030. There were 240 admissions, 79 recoveries, and 117 deaths. The recoveries stand at 32·9, and the deaths at 11·4 per cent. Hereditary influence is the assigned cause of the insanity in 75 of the cases admitted, and intemperance in drink accounts for 15. Forty-four of the deaths were owing to brain diseases or maniacal exhaustion, and 10 to consumption. The Commissioners say the asylum is full on the males' side and crowded on the female side. The population of the asylum has increased at the rate of about 50 a year, and as it already contains over a thousand patients, it is quite certain that no further enlargement should be thought of. Indeed, the visitors say "it may be considered advisable to alter and amend the original agreement between the three counties," and we hope this may be construed to mean that the partnership will be dissolved and a new asylum built. It is most gratifying to note that the Matron and Head Nurse both received pensions—the former £100 a year, and the latter £50. The visitors thank Dr. Swain for "his able performance of his numerous duties."

The rate of maintenance is 8s. 1d. per head per week.

### NORTH RIDING ASYLUM, YORK.

An increase in the number of patients from 686 to 705 has to be noted. The admissions were 180, the recoveries 74, and the deaths 69. The percentage of recoveries is 43·5, and that of the deaths is 10. Hereditary influence accounts for 16 of the admissions, and drink for 15; but Table 10 states that the cause was unknown in 67. Brain diseases account for 37 of the deaths, and consumption for 10. Mr. Hingston had an experience during the year which fortunately has rarely, or perhaps never, occurred in any other asylum. "Much inconvenience was occasioned," he says, "from the exceptionally high flood which came over the embankment in October last, inundating the kitchen garden and rising several feet within the main building."

The weekly cost per head is 9s. 3⅝d.

